# General anesthesia exposure and risk of dementia: a meta-analysis of epidemiological studies

**DOI:** 10.18632/oncotarget.19524

**Published:** 2017-07-24

**Authors:** Jingjing Jiang, Yunxia Dong, Wei Huang, Min Bao

**Affiliations:** ^1^ Department of Anesthesiology, Shengjing Hospital of China Medical University, Shenyang, China; ^2^ Department of Neurosurgery, Shengjing Hospital of China Medical University, Shenyang, China

**Keywords:** general anesthesia, dementia, epidemiological studies, meta-analysis

## Abstract

The association between exposure to general anesthesia and dementia risk has been inconsistently reported across epidemiological studies. To better understand the association, we conducted a meta-analysis of epidemiological studies. PubMed and Embase were searched through April 2017. Random-effects models were used to pool association estimates. We further evaluated potential dose-response relationship. Based on literature search, seven prospective/cohort studies, 11 case-control studies, and a pooled analysis of six case-control studies were identified. Sixteen of these studies were with high quality. After pooling available risk estimates, overall no significant association between exposure to general anesthesia (yes versus no) and dementia risk was detected (odds ratio (OR) = 1.03, 95% confidence interval (CI) 0.90–1.19, *p* for heterogeneity < 0.001). The null association persisted in the majority of subgroup analyses, although a significant positive association was detected in studies collecting anesthesia exposure using records (OR = 1.22, 95% CI 1.01–1.47, *p* for heterogeneity < 0.001), a method that is less prone to bias compared with interview or questionnaire using proxy reporters. Based on the dose-response analysis of three studies, a significant nonlinear relationship between times of exposure to general anesthesia and increased risk of dementia was suggested (*p* < 0.0001). Overall, this meta-analysis suggests that overall the evidence from epidemiological studies supporting a link between general anesthesia exposure and an increased dementia risk is not very strong, while an association was suggested in the studies collecting anesthesia exposure using records and those providing anesthesia exposure frequency data. Further well-designed studies are warranted to better characterize the relationship of interest.

## INTRODUCTION

Worldwide, dementia represents a huge public health burden [1]. In 2010, there were approximately 35.6 million people living with dementia, and the number is expected to be increased to 65.7 million in 2030 [1]. Dementia is a challenging disease because it is progressive and irreversible. Health care expenditures among individuals with dementia have been estimated to be significantly higher than those for other common diseases including heart disease and cancer [2]. It is critical to better understand its etiology and risk factors, at the aim for developing appropriate strategies to decrease its incidence and public health burden. Although research has suggested that age, sex, smoking, alcohol drinking, education, physical activity, body mass index, diabetes, hypertension, atherosclerosis and certain genetic factors may be associated with risk of dementia [3–7], the relationship with other factors has not been fully characterized.

As the most common form of dementia, Alzheimer's disease (AD) constitutes about 50–56% of dementia patients [8, 9]. The pathophysiology of AD is thought to involve an accumulation of β-amyloid peptide (Aβ) and neurofibrillary tangles that are composed of abnormally hyperphosphorylated and aggregated form of tau proteins [9]. Interestingly, anesthesia, which frequently accompanies surgery, is suggested to be potentially related to neurodegenerative complications. For example, basic research showed that inhaled anesthetics could promote the formation of Aβ plaques and neurofibrillary tangles [10–14]. Exposure to anesthetic drugs has also been reported to impair long-time memory based on animal research [15]. Despite these reports, however, findings from human studies have not been consistent. In several observational studies, exposure to general anesthesia was observed to be significantly associated with an increased risk of dementia/AD [16–20]. On the other hand, such an association was not detected in some other studies [21–28]. In three prospective studies, significant inverse associations with decreased dementia/AD risk were detected [29, 30]. Considering that different studies may vary for the study design, number of participants, as well as analysis method, it is important to critically assess all available evidence including evaluating the heterogeneity across studies to better understand the exact relationship between exposure to general anesthesia and dementia risk.

Seitz et al. summarized available epidemiological studies up to April 2010 for assessing the association between general anesthesia exposure and AD risk, which detected a null association [31]. It is worth noting that, however, studies evaluating an association with risk of more general dementia (beyond the scope of AD) were not evaluated in this meta-analysis [30]. Furthermore, since the conduction of this meta-analysis, multiple additional studies evaluating the association of interest have been published [16, 18, 21, 22, 24]. To better characterize the relationship between general anesthesia exposure and risk of dementia beyond merely AD, we performed a comprehensive meta-analysis of all available epidemiological studies up to April 2017. We also performed dose-response analyses to better understand potential dose-response relationship between the frequency of general anesthesia and dementia risk.

## RESULTS

### Literature search and study characteristics

**Figure 1:**
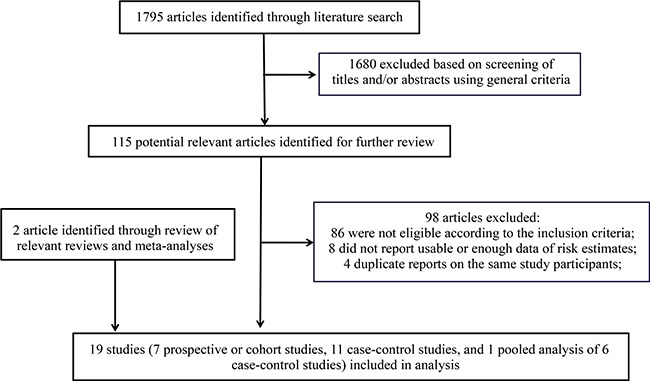
Flow chart for selection of eligible studies

Our literature search and article screening processes are shown in Figure 1. Briefly, 1,795 articles were identified through the literature search. After title/abstract screening using pre-defined criteria, 1,680 articles were excluded. We fully assessed the remaining 115 articles for their contents. Among them, 98 were further excluded due to 1) not eligible according to the pre-defined inclusion criteria; 2) did not report usable or sufficient data of risk estimates; or 3) duplicated reports containing same participants with larger studies. We identified two additional eligible studies by reviewing the reference lists of relevant review articles and meta-analyses [29]. Overall, 19 studies, including seven prospective or cohort studies, 11 case-control studies, and a pooled analysis of six case-control studies meeting the inclusion criteria were included in our meta-analysis [16–30, 32–34]. The detailed characteristics of these studies are shown in Supplementary Table 1. Briefly, nine were conducted in America [18, 19, 21, 22, 25, 27, 29, 33], five were conducted in Europe [17, 20, 23, 28, 30], three were conducted in Asia [16, 24, 26], one was conducted in Europe-Asia (Turkey) [32], and one was an international study (the pooled analysis) [34]. Eight of these studies collected information of exposure to general anesthesia by reviewing relevant records; and other studies collected exposure information using interview or questionnaire, which is thought to be more prone to biases, especially for case-control studies in which proxy reporters were often used. The quality ratings for these studies are shown in Table 1 and Table 2. Overall, 16 studies were classified as high-quality studies and three studies were categorized as low-quality studies.

**Table 1:** Quality assessment of included prospective or cohort studies using the Newcastle-Ottawa quality assessment scale

Study	Exposed cohort represents average in community	Selection of the non-exposed cohort from same community	Ascertain exposure through records or structured interviews	Demonstrate that outcome not present at study start	Exposed and non-exposed matched and/or adjusted by factors	Ascertain outcome via independent blind assessment or record linkage	Follow-up long enough for outcome to occur	Loss to follow-up < 20%	Overall Score
Plassman, 2009	1	1	1	0	1	1	1	1	7
Aiello Bowles, 2016	1	1	1	1	2	1	1	1	9
Ritchie, 2010	1	1	1	1	2	1	1	1	9
Lee, 2005	0	1	1	1	2	1	1	1	8
Vanderweyde, 2010, Hernia Study	1	1	0	0	2	0	1	1	6
Vanderweyde, 2010, Prostate Study	1	1	0	0	2	0	1	1	6
Yip, 2006	1	1	1	0	2	1	1	1	8

**Table 2:** Quality assessment of included case-control studies using the Newcastle-Ottawa quality assessment scale

Study	Case defined with independent validation	Representativeness of the cases	Selection of controls from community	Statement that controls have no history of outcome	Cases and controls matched and/or adjusted by factors	Ascertain exposure by secure record/blinded structured interview	Same method of ascertainment for cases and controls	Same response rate for both groups	Overall Score
Amaducci, 1986	1	1	0	1	2	1	1	1	8
Li, 1992	1	1	1	1	2	1	1	1	9
CSHA, 1994	1	1	1	0	2	0	1	1	7
Bohnen, 1994	1	1	1	1	2	1	1	1	9
Gasparini, 2002	1	1	0	0	2	1	1	1	7
Harmanci, 2003	1	1	1	0	2	1	1	1	8
Zuo, 2010	1	1	0	0	2	1	1	1	7
Sprung, 2013	1	1	1	1	2	1	1	1	9
Bufill, 2009	1	1	1	0	2	1	1	1	8
Chen, 2014	1	1	1	0	2	1	1	1	8
Tsuda, 2015	1	1	0	0	0	1	1	1	5
Breteler, 1991-ref2	1	0	1	0	2	1	1	1	7
Breteler, 1991-ref7	1	1	0	1	2	1	1	1	8
Breteler, 1991-ref8	1	1	0	0	2	1	1	1	7
Breteler, 1991-ref9	1	1	1	1	2	1	1	1	9
Breteler, 1991-ref31	1	1	1	0	2	1	1	1	8
Breteler, 1991-ref32	1	1	1	1	2	1	1	1	9

### Exposure to general anesthesia and risk of dementia

**Figure 2:**
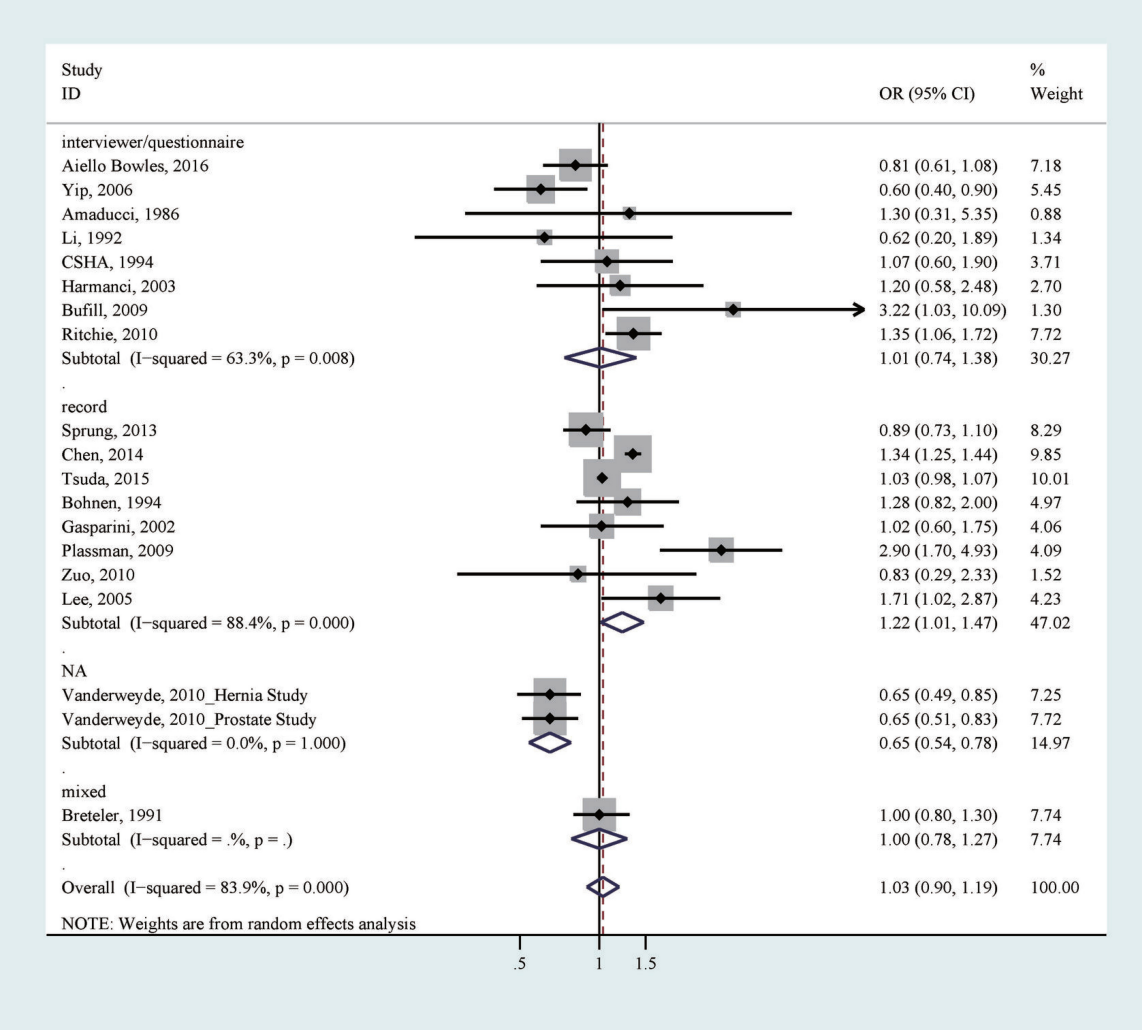
The association between any exposure to general anesthesia and risk of dementia

All 19 studies reported the association between any exposure to general anesthesia (yes versus no) and dementia risk [16–30, 32–34]. After pooling all association estimates, we did not detect a significant association (odds ratio (OR) = 1.03, 95% confidence interval (CI) = 0.90–1.19), with considerable heterogeneity (*I*^2^ = 83.9%; *p* for heterogeneity < 0.001; Table 3, Figure 2). We did not detect apparent publication bias by Egger's test (*p* for bias: 0.807) and Begg's test (*p* for bias: 0.234). Sensitivity analysis revealed that the 19 study-specific ORs (95% CIs) ranged from as low as 1.01 (0.87–1.17) (*I*^2^ = 73.3%) after omitting the study by Chen et al. to as high as 1.05 (0.87–1.27) (*I*^2^ = 82.8%) after omitting the study by Tsuda et al. According to the Galbraith plot, five studies contributed considerably to the heterogeneity [16, 19, 29, 30]. After excluding these studies from the analysis, a marginally positive association was suggested (OR = 1.03, 95% CI 0.99–1.07), with no considerable heterogeneity (*I*^2^ = 32.1%; *p* for heterogeneity: 0.119). We further performed subgroup analyses according to study design, study quality, location, method of exposure collection, type of controls, number of cases (< 250 or ≥ 250), publication year (earlier than 2010 or 2010−), and adjustment of age, sex or education (Table 3). Even though the null association persisted in the majority of the tested strata, a significant association between ever exposure to general anesthesia and an increased risk of dementia was detected in studies using record to collect exposure information (OR = 1.22, 95% CI = 1.01–1.47; *p* for heterogeneity < 0.001; Table 3; Figure 2), a method thought to be less prone to biases.

**Table 3:** Summary risk estimates of the association between exposure to general anesthesia and risk of dementia

	No. of reports	OR (95% CI)	*I*^2^	*P* for heterogeneity
**Overall**	19	1.03 (0.90–1.19)	83.9%	< 0.001
**Subgroup analysis**				
**Study design**				
Prospective/cohort	7	1.01 (0.71–1.44)	88.4%	< 0.001
Case-control	12	1.09 (0.94–1.27)	78.0%	< 0.001
**Study quality**				
High	16	1.14 (0.96–1.36)	73.2%	< 0.001
Low	3	0.77 (0.53–1.11)	91.2%	< 0.001
**Location**				
America	9	1.02 (0.78–1.34)	79.8%	< 0.001
Europe	5	1.12 (0.70–1.81)	72.9%	0.005
Asia	3	1.14 (0.88–1.47)	95.1%	< 0.001
Europe-Asia	1	1.20 (0.58–2.48)	-	-
International	1	1.00 (0.80–1.30)	-	-
**Sources of exposure collection**				
Record	8	**1.22 (1.01–1.47)**	88.4%	< 0.001
Questionnaire or interview	8	1.01 (0.74–1.38)	63.3%	0.008
**Type of controls**				
Population-based	7	1.16 (0.91–1.48)	67.0%	0.006
Hospital-based	3	1.02 (0.98–1.07)	0	0.924
**Number of cases**				
< 250	8	1.05 (0.75–1.45)	65.5%	0.005
≥ 250	8	1.00 (0.85–1.17)	87.9%	< 0.001
**Study publication time**				
Earlier than 2010	11	1.22 (0.91–1.63)	66.3%	0.001
2010–	8	0.94 (0.79–1.12)	91.4%	< 0.001
**Estimate adjusted for age**				
Yes	16	0.99 (0.81–1.19)	82.9%	< 0.001
No	3	1.50 (0.75–2.98)	86.4%	0.001
**Estimate adjusted for sex**				
Yes	12	1.03 (0.85–1.25)	75.1%	< 0.001
No	6	1.21 (0.86–1.70)	82.8%	< 0.001
**Estimate adjusted for education**				
Yes	3	0.77 (0.57–1.04)	33.9%	0.22
No	16	1.09 (0.93–1.26)	84.9%	< 0.001

### Dose-response analysis

**Figure 3:**
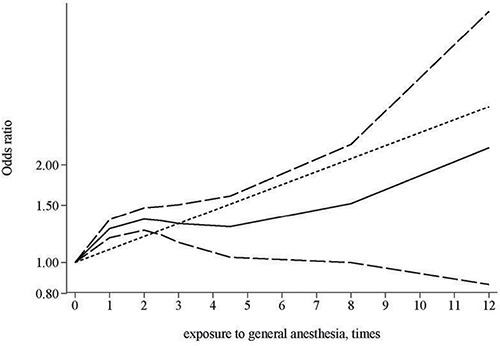
Dose-response relationship between times of exposure to general anesthesia and risk of dementia

Three studies provided association estimates for frequency of general anesthesia exposure (times) and risk of dementia [16, 22, 27]. We analyzed data of these three studies to evaluate the dose-response relationship between general anesthesia exposure frequency and risk of dementia. Assuming a non-linear relationship, the dose-response analysis suggested a significant relationship (*p* < 0.0001; Figure 3). The test for nonlinearity suggested that a nonlinear relationship might be more appropriate compared with a linear relationship (*p* for nonlinearity: 0.0001).

## DISCUSSION

We performed a comprehensive meta-analysis of observational studies to evaluate the association between exposure to general anesthesia and dementia risk. After summarizing all available evidence, overall no significant association of ever general anesthesia exposure was detected. We observed a positive association in studies collecting exposure using records, a method with less possibility of prone to biases. We also noted a significant nonlinear dose-response relationship between times of exposure to general anesthesia and an increased risk of dementia, based on data from three studies with relevant data. Further well-designed studies are warranted to better characterize the relationship between exposure to general anesthesia and dementia risk.

Following surgery and anesthesia, short-term cognitive dysfunction has been frequently observed. Such postoperative cognitive dysfunction (POCD) [35] has been speculated to be potentially related to anesthesia [36]. It has been identified that general anesthetics can produce both neurotoxicity and cognitive impairment in animals [37], although the association in humans has been inconsistently reported [36]. With regards to dementia/AD, a potential link with anesthesia exposure has been implicated in basic lab research as discussed in the introduction, and human biomarker studies. In patients undergoing coronary artery bypass surgery, at six months cerebrospinal fluid (CSF) tau and Aβs levels were significantly increased and decreased, respectively [38]. These changes are consistent with patterns in AD development. In another study, it was observed that after anesthesia and surgery, the CSF total-tau/Aβ_1–42_ ratio and proinflammatory cytokines including IL-6, TNF-α, and IL-10 were elevated [39], suggesting an elicited neuroinflammatory response. Aligned with these findings, we detected a significant dose-response relationship between general anesthesia exposure and increased dementia risk, albeit this is based on merely three studies with relevant data. In studies evaluating the association of ever exposure to general anesthesia, a positive association was detected in studies collecting exposure using records, but not in overall studies or subgroups of studies stratified by other assessed characteristics. Further research, especially those evaluating the dose response relationship of exposure to general anesthesia, with exposure collected using records, would be necessary to clarify the validity of our findings.

Our study has several strengths. To our knowledge, to date this is the most comprehensive meta-analysis evaluating the association between general anesthesia exposure and risk of dementia. Besides studies assessing an association with AD risk, we also included studies evaluating associations with dementia beyond merely AD in our quantitative synthetization, which is for the first time, to the best of our knowledge. We carefully performed sensitivity analysis and subgroup analyses based on pre-defined variables. Besides evaluating the association of ever exposure to general anesthesia, we performed dose-response analyses to further understand the relationship of interest. Our findings suggest a need of future research for better characterizing the relationship of interest.

We also need consider several potential limitations of our study for an appropriate interpretation of our results. First, we cannot rule out the possibility that the association estimates used in the current meta-analysis may not be fully adjusted for relevant covariates. For example, age, sex, education and vascular risk factors such as hypertension, diabetes, smoking and heart disease are known risk factors for dementia [40]. However, not all of the included studies in our meta-analyses sufficiently adjusted for all these known covariates, especially for education and vascular risk factors. Residual confounding may thus be an issue. In our meta-analysis we performed subgroup analyses carefully assessing whether adjustment of important covariates may influence the association results. Second, differences in the assessment of general anesthesia exposure across the included studies could be an important source of heterogeneity. In eight studies the exposure information was collected using medical or other records, while in eight other studies it was collected using questionnaire or interview. The method of questionnaire or interview is particularly prone to biases in case-control studies, in which proxy reporters were often used for collecting relevant information. Indeed, in three studies, the accuracy of anesthesia exposure collected through proxy reporters was evaluated by comparing a history provided by controls with normal cognition with information provided by proxy subjects of these controls, and it was identified that the agreement was only in moderate range [25, 41, 42], suggesting that such a method may be prone to biases. Third, there was high heterogeneity across the included studies in our meta-analysis. The heterogeneity tended not to disappear in our subgroup analyses according to study design, quality, location, method of exposure collection, type of controls, number of cases, publication year, and adjustment of key covariates. The several studies contributing to the high heterogeneity were detected by Galbraith plot. Finally, our findings were based on epidemiological studies, and thus the identified associations may not necessarily infer causation, due to the observational nature of such studies. Future well-designed studies resolving the above-mentioned limitations would be needed to better understand the relationship of interest.

In conclusion, after summarizing available evidence from epidemiological studies, there is no strong evidence suggesting a significant association between ever exposure to general anesthesia and an increased dementia risk. However, in studies collecting exposure using records, such an association cannot be excluded. There is also a suggested nonlinear dose-response relationship between times of general anesthesia exposure and increased risk of dementia, although the evidence is based only on three available studies. Further large scale, well-designed studies are needed to better clarify the relationship between general anesthesia exposure and dementia risk.

## MATERIALS AND METHODS

### Data sources and search strategies

A literature search of PubMed and Embase was conducted through April 2017 to identify eligible epidemiological studies. We used the following search keywords: (Alzheimer OR Dementia) AND (anesthesia OR anesthetic). No language or study sample size restriction was applied. Furthermore, we manually reviewed the reference lists of dozens of relevant review and meta-analysis articles aiming to identify additional eligible studies.

### Study selection

The following criteria were used to determine eligible studies, as: (1) prospective/retrospective cohort studies, case-cohort studies, case-control studies, or pooled analysis of cohort or case-control studies, (2) assessed the association between exposure to general anesthesia and risk of dementia (including AD), and (3) presented OR, relative risk (RR), or hazard ratio (HR) estimates with 95% CIs or sufficient data for determination. If we identified multiple publications containing same participants, we retained the one with the largest number of cases in our meta-analysis.

### Data abstraction and quality assessment

Two investigators independently conducted the title/abstract screening, full-text screening, data extraction, as well as quality assessment. Disagreements were resolved by discussion with other investigators. The following data were abstracted from each study, as: the first author's last name, study publication year, study country/region, study design, as well as characteristics of the study sample (sample size, age, and association estimates). If multiple estimates of the same association were available, the one with an adjustment for the most appropriate covariates was used.

We used the Newcastle-Ottawa Quality Assessment Scale [43] to assess the quality of included studies. Such a scale assesses aspects regarding population/sampling methods, exposure/outcome collections, as well as statistical matching/adjustments of the data. Quality scores were assigned for each aspect, and an overall score was assigned for each study, with a maximum possible score of 9. Studies with a score of ≥ 7 were categorized as high-quality studies, and those with a score of < 7 were categorized as low-quality studies.

### Statistical methods

We synthesized all available association estimates between any exposure to general anesthesia and risk of dementia. When possible, we tried to convert estimates of RRs or HRs into ORs. There were situations that such a conversion was not possible due to a lack of sufficient data, and for relevant studies, since the proportion of dementia development was low (< 10%), RRs and HRs were deemed equivalent to ORs. *I*^2^ was used to evaluate heterogeneity across studies [44, 45]. Considering that the included studies differ significantly with each other, the random-effects model was used to pool the log-transformed ORs [46]. We conducted sensitivity analysis excluding one study at a time to assess whether any specific study considerably influenced the overall pooled results. We also performed subgroup analyses to better understand the relationship of interest within strata defined by study design, quality, location, exposure collection method, type of controls, number of cases (≥ 250 or < 250), study publication year (before 2010 or 2010-), and important dementia risk factors age, sex and education. To investigate potential publication bias, Egger's test [47] and Begg's test [48] were performed. A *p* value threshold of 0.05 was used to determine whether there was significant publication bias.

We explored potential dose-response relationship between times of exposure to general anesthesia and risk of dementia [49]. We used the method by Greenland et al. [49] to determine study-specific slopes (linear trends) and 95% CIs from the natural logs of the ORs and CIs across categories of times of exposure to general anesthesia. For such analysis, the number of cases, overall subjects, ORs, and 95% CIs for at least three exposure categories were needed. We set the midpoint of each category of times of exposure to general anesthesia by averaging the lower and upper bounds. When the highest category of general anesthesia exposure did not reported an upper bound, we assumed the open ended interval of the highest category was the same with that of the second highest category [50, 51]. We examined potential nonlinear dose-response relationship using fractional polynomial models with restricted cubic splines and four knots at fixed percentiles (5%, 35%, 65%, and 95%) of the distribution [39]. We conducted a likelihood ratio test to determine whether a nonlinear or linear relationship was more appropriate. All statistical analyses were performed using Stata 12.1 software (StataCorp, College Station, TX, USA).

## SUPPLEMENTARY MATERIALS TABLES




