# Supplementary material for: General anesthesia exposure and risk of dementia: a meta-analysis of epidemiological studies
Source: Oncotarget. 2017 Jul 24;8(35):59628–37. doi: 10.18632/oncotarget.19524 (PMC5601762; doi:10.18632/oncotarget.19524)
Supplement: Supplementary file 1 [file oncotarget-08-59628-s001.pdf]

General anesthesia exposure and risk of dementia: a meta-analysis of epidemiological studies

SUPPLEMENTARY MATERIALS

Supplementary Table 1: Characteristics of included studies assessing associations of general anesthesia exposure and risk of dementia. See Supplementary_Table_1
