# Supplementary material for: General anesthesia exposure and risk of dementia: a meta-analysis of epidemiological studies
Source: Oncotarget. 2017 Jul 24;8(35):59628–37. doi: 10.18632/oncotarget.19524 (PMC5601762; doi:10.18632/oncotarget.19524)
Supplement: Supplementary file 2 [file oncotarget-08-59628-s002.docx]

**Supplementary** **Table 1. Characteristics of included studies assessing associations of general anesthesia exposure and risk of dementia**

| **Author, study region** | **Study type** | **Cases/subject or control (age), follow up duration** | **Exposure categories (exposure/case assessment)** | **OR (95%CI)** | **Matched or adjusted factors** |
| --- | --- | --- | --- | --- | --- |
| Amaducci, 1986, northern Italy | PC-CS/HC-CS | 116/213 (40-80) | Exposure to general anesthesia  No  Yes  (trained interviewer/clinical diagnosis) | 1.0 (ref)  1.30 (0.31-5.35) | age, sex, and region of residence |
| Li, 1992, China | PC-CS | 70/140 (mean 65) | Exposure to general anesthesia  No  Yes  (trained interviewer/clinical diagnosis by psychiatrists using criteria of NINCDS/ADRDA) | 1.0 (ref)  0.62 (0.20-1.89) | age, sex |
| CSHA, 1994, Canada | PC-CS | 258/535 (65+) | Exposure to general anesthesia  No  Yes  (trained interviewer or questionnaire/clinical diagnosis using criteria of NINCDS/ADRDA) | 1.0 (ref)  1.07 (0.60-1.90) | age group, study center, and residence in community or institution |
| Bohnen, 1994, Rochester, MN, US | PC-CS | 252/252 (NA) | Exposure to general anesthesia  No  Yes  (medical record review /medical record review) | 1.0 (ref)  1.28 (0.82-2.00) | age, sex |
| Gasparini, 2002, Roma, Italy | HC-CS | 115/230 (mean 69) | Exposure to general anesthesia  No  Yes  (hospital records/clinical diagnosis using criteria of NINCDS/ADRDA) | 1.0 (ref)  1.02 (0.60-1.75) | sex, age (±3 years) and geographic area of residence |
| Harmanci, 2003, Istanbul, Turkey | PC-CS | 57/127 (70+) | Exposure to general anesthesia  No  Yes  (trained interviewer/clinical diagnosis using the National Institute of Neurologic and Communicative  Disorders and Stroke and the Alzheimer’s Disease  and Related Disorders Association criteria) | 1.0 (ref)  1.20 (0.58-2.48) | level of education, Use of electricity for residential heating, occupational group |
| Plassman, 2009, US | CS | 856 subjects overall (70+) | Exposure to general anesthesia  No  Yes  (Medicare record review/clinical and neuropsychological assessment) | 1.0 (ref)  2.90 (1.70-4.93) | number of hospitalizations prior to the outcome date |
| Zuo, 2010, Virginia, US | HC-CS | 26/161 (mean 76 for cases, 44 for controls) | Exposure to general anesthesia  No  Yes  (Medicare record review/clinical diagnosis using ICD-9-CM codes) | 1.0 (ref)  1.267 (0.519-3.094) | Age, sex, length of hospital stay, length of operating room time |
| Aiello Bowles, 2016, Washington state | CS | 752 for AD & 946 for dementia/3988 (65+), mean 7 years | Exposure to general anesthesia  No  Yes  (trained interviewer/ diagnosis using criteria of DSM-4 or NINCDS/ADRDA) | 1.0 (ref)  0.81 (0.61-1.08) | Adult Changes in Thought study cohort, age, age at study entry, sex, education, hypertension, diabetes mellitus, smoking, stroke, coronary heart disease, body mass index, exercise, self-rated health, depression, Parkinson’s disease, Charlson Comorbidity Index, difficulty with activities of daily living |
| Sprung, 2013, Rochester, MN, US | PC-CS | 877/877 (mean ~82) | Exposure to general anesthesia  No  Yes  (medical record review/ medical record review using criteria of DSM-4 or NINCDS/ADRDA) | 1.0 (ref)  0.89 (0.73/1.10) | age, sex |
| Breteler, 1991, international | 6 case-control studies (PC-CS & HC-CS) | 1925/1952 | Exposure to general anesthesia  No  Yes | 1.0 (ref)  1.0 (0.8-1.3) | age, sex |
| Bufill, 2009, Spain | PC-CS | NA/NA (80+) | Exposure to general anesthesia  No  Yes  (interview/examination using NINCDS-ADRA criteria) | 1.0 (ref)  3.22 (1.03-10.09) | age, sex |
| Ritchie, 2010, Montpellier, France | CS | 31/1433 (mean 72.5), median 7.31 years | Exposure to general anesthesia  No  Yes  (trained interviewer/neurologist examination using interview including cognitive testing) | 1.0 (ref)  1.35 (1.06-1.72) | age, sex |
| Lee, 2005, US | Retrospective CS | 119/9170 (55+), mean ~5.5 years | Exposure to general anesthesia  No  Yes  (medical record/clinical diagnosis using ICD-9 code) | 1.0 (ref)  1.71 (1.02-2.87) | age, number of surgeries, number of diagnoses, and length of stay for index hospitalization |
| Vanderweyde, 2010, Hernia Study | CS | ~218/3769 (mean ~70), mean 4.5 years | Exposure to general anesthesia  No  Yes  (NA/NA) | 1.0 (ref)  0.65 (0.49-0.85) | age, length of stay, number of procedures, and number of diagnoses during the index  hospitalization |
| Vanderweyde, 2010, Prostate Study | CS | NA/6511 (mean ~70), | Exposure to general anesthesia  No  Yes  (NA/NA) | 1.0 (ref)  0.65 (0.51-0.83) | age, length of stay, number of procedures, and number of diagnoses during the index  hospitalization |
| Yip, 2006, UK (England and Wales) | Nested case-control study | 275/3800 (65+) | Exposure to general anesthesia  No  Yes  (trained interviewer/assessment interview using DSM-III-R) | 1.0 (ref)  0.6 (0.4-0.9) | age, sex, education and social class |
| Chen, 2014, Taiwan | PC-CS | 5345/21380 (50+) | Exposure to general anesthesia  No  Yes  (record /clinical diagnosis using ICD-9 code) | 1.0 (ref)  1.34 (1.25-1.44) | age, gender, and index date |
| Tsuda, 2015, Japan | HC-CS | 9419/78226 (70+) | Exposure to general anesthesia  No  Yes  (record /clinical diagnosis using ICD-10 code) | 1.0 (ref)  1.03 (0.98-1.07) | NA |

CS: cohort study; PC-CS: population-based case-control study; HC-CS: hospital-based case-control study; OR: odds ratio; CI: confidence interval; Ref: reference; NA: not available
